# Transcriptomic Analysis of Calcium Remodeling in Colorectal Cancer

**DOI:** 10.3390/ijms18050922

**Published:** 2017-04-27

**Authors:** Enrique Pérez-Riesgo, Lucía G. Gutiérrez, Daniel Ubierna, Alberto Acedo, Mary P. Moyer, Lucía Núñez, Carlos Villalobos

**Affiliations:** 1 Institute of Molecular Biology and Genetics (IBGM), National Research Council (CSIC), 47003 Valladolid, Spain; epercamh@gmail.com (E.P.-R.); gonzalez.gutierrez.lucia@gmail.com (L.G.G.); d.ubierna@gmail.com (D.U.); nunezl@ibgm.uva.es (L.N.); 2 Department of Biochemistry and Molecular Biology and Physiology, University of Valladolid, 47005 Valladolid, Spain; 3 AC-Gen Reading Life, 47011 Valladolid, Spain; acedo@biomemakers.com; 4 INCELL Corporation, San Antonio, TX 78249, USA; mpmoyer@incell.com

**Keywords:** colorectal cancer, Ca^2+^ remodeling, transcriptomics, RNA-sequencing

## Abstract

Colorectal cancer (CRC) cells undergo the remodeling of intracellular Ca^2+^ homeostasis, which contributes to cancer hallmarks such as enhanced proliferation, invasion and survival. Ca^2+^ remodeling includes critical changes in store-operated Ca^2+^ entry (SOCE) and Ca^2+^ store content. Some changes have been investigated at the molecular level. However, since nearly 100 genes are involved in intracellular Ca^2+^ transport, a comprehensive view of Ca^2+^ remodeling in CRC is lacking. We have used Next Generation Sequencing (NGS) to investigate differences in expression of 77 selected gene transcripts involved in intracellular Ca^2+^ transport in CRC. To this end, mRNA from normal human colonic NCM460 cells and human colon cancer HT29 cells was isolated and used as a template for transcriptomic sequencing and expression analysis using Ion Torrent technology. After data transformation and filtering, exploratory analysis revealed that both cell types were well segregated. In addition, differential gene expression using R and bioconductor packages show significant differences in expression of selected voltage-operated Ca^2+^ channels and store-operated Ca^2+^ entry players, transient receptor potential (TRP) channels, Ca^2+^ release channels, Ca^2+^ pumps, Na^+^/Ca^2+^ exchanger isoforms and genes involved in mitochondrial Ca^2+^ transport. These data provide the first comprehensive transcriptomic analysis of Ca^2+^ remodeling in CRC.

## 1. Introduction

Ca^2+^ signaling is involved in the control of a large series of cell and physiological functions in health and disease from exocytosis and muscle contraction to gene expression, cell proliferation, migration and cell death [1]. Over the last few years, evidence has been accumulating that dis-homeostasis of intracellular Ca^2+^ may be involved in many different disorders including cancer [2,3]. In support of this view, several authors have reported that remodeling of Ca^2+^ signaling in different forms of cancer contributes to cancer hallmarks, including exaggerated cell proliferation, acquisition of cell migration and invasion capabilities, and enhanced resistance to cell death [2,3]. We have recently compared intracellular Ca^2+^ homeostasis in normal human colonic cells and human colon carcinoma cells [4]. We found critical differences that may contribute to several of the above cancer hallmarks. The most salient characteristics of Ca^2+^ remodeling in colon cancer cells relative to their normal counterparts are the dramatic rise in store-operated Ca^2+^ entry (SOCE) and the partial depletion of intracellular Ca^2+^ stores [4]. Enhanced entry of Ca^2+^ in colon cancer cells correlates with enhanced cell proliferation and all SOCE antagonists tested and/or targeting molecular players involved in this pathway result in inhibition of cell proliferation and cell migration [4,5,6]. In addition, the partial depletion of Ca^2+^ stores provides resistance to cell death, probably because of impaired mitochondrial Ca^2+^ overload and apoptosis [4,5,6]. 

SOCE, first reported by James W Putney in 1986 [7,8] is a ubiquitous Ca^2+^ entry pathway activated after agonist-induced release of Ca^2+^ from intracellular stores at the endoplasmic reticulum (ER). This is due to the opening of intracellular Ca^2+^ release channels gated by phospholipase C-dependent synthesis of inositol trisphosphate IP_3_ receptors (IP_3_Rs) and/or Ca^2+^-induced Ca^2+^ release mediated by ryanodine receptors (RYRs). At the molecular level, it is well established that Stromal Interaction Molecules (STIM) sense ER Ca^2+^ levels. Emptying of Ca^2+^ stores promotes STIM oligomerization at the ER endomembranes [9] and their interaction with cation channels in the plasma membrane of the Orai1 [10] and transient receptor potential (TRP) families of Ca^2+^ channels [11]. Activation of SOCE not only permits the refilling of Ca^2+^ stores but also promotes a sustained increase in intracellular Ca^2+^ concentration that is critical for the sustained activation of effector proteins including, for instance, calcineurin and the ensuing activation of the nuclear factor of activated T cells [10]. 

After stimulation, the increased cytosolic free Ca^2+^ concentrations ([Ca^2+^]_cyt_) must return to resting levels in the low nM range. This is achieved by systems that transport Ca^2+^ back from cytosol to the extracellular space or into the ER against a large electrochemical gradient for Ca^2+^. This is carried out by different adenosine triphosphate (ATP)ase Ca^2+^ pumps including plasma membrane Ca^2+^ ATPases (PMCAs) [12], sarcoplasmic and/or endoplasmic reticulum Ca^2+^ ATPases (SERCAs) [13] and the secretory pathway Ca^2+^ ATPases (SPCAs) [14]. An additional cotransporter, the Na^+^/Ca^2+^ exchanger, uses the passive transport of Na^+^ into the cell to extrude Ca^2+^ out of the cell [15]. Finally, as has become increasingly evident in recent years, mitochondria contribute to clear Ca^2+^ loads by means of the mitochondrial Ca^2+^ uniporter (MCU) and regulatory proteins [16]. In this case, Ca^2+^ is transported down a huge electromotive force, the mitochondrial potential (Δ*Ψ*) of about −180 mV, negative inside the mitochondrial matrix, that enables Ca^2+^ influx into mitochondria [17,18]. This potential is particularly high in cancer cells due to the Warburg effect, the metabolic signature of cancer cells [19]. Interestingly, mitochondria are also involved in control of SOCE in different cell types [20,21], including colorectal cancer cells [18,22] where, store-operated Ca^2+^ channels may undergo slow, Ca^2+^-dependent inactivation unless this process is prevented by Ca^2+^ removal by surrounding mitochondria [20,21]. 

Our recent analysis of Ca^2+^ remodeling in colon cancer cells revealed increased expression of several molecular players involved in SOCE [4,5,6]. However, Ca^2+^ remodeling is complex and may involve changes in expression and/or activity of hundreds of genes related to Ca^2+^ signaling [23], with nearly 80 of them directly involved in Ca^2+^ transport across biological membranes. By taking advantage of Next Generation Sequencing technologies, particularly RNA-sequencing (RNA-seq) by Ion Torrent methodologies, we were able to study in detail intracellular Ca^2+^ homeostasis in NCM460 cells reflecting normal human colonic cells [24,25] and HT29 cells [26] representing human colorectal cancer cells, to investigate, for the first time, the transcriptomics of Ca^2+^ remodeling in CRC. To this end, mRNA was isolated from four independent cultures of NCM460 and four independent cultures of HT29 cells to be used as templates for transcriptomic analysis of the selected 77 genes shown in Table 1. 

The gene transcripts studied include all ten voltage-operated Ca^2+^ channels (VOCCs); all seven well-established players involved in SOCE; all 27 TRP channels; all six Ca^2+^ release channels—including IP_3_ receptors and ryanodine receptor isoforms—all nine Ca^2+^ pumps including PMCAs, SERCAs and SPCAs; the three Na^+^/Ca^2+^ exchanger isoforms; all known genes involved in mitochondrial Ca^2+^ transport; and other related proteins. Clustering analysis segregated data in two different cell populations and genes were segregated into six independent gene families according to expression behavior. Overall, we found that 30 genes are expressed differentially in CRC cells relative to normal cells, thus providing a first comprehensive view of Ca^2+^ remodeling in CRC.

## 2. Results and Discussion

### 2.1. Data Set Transformation and Filtering

mRNA from four independent cultures of normal (NCM460) human colonic cells and four independent cultures of human colorectal (HT29) cells were isolated and used as templates for transcriptomic analysis of 77 genes involved in intracellular Ca^2+^ transport (Table 1) as detailed in the Methods section. 

RNA-seq data sets consist of aligned reads from gene fragmentations against a reference genome. Accordingly, the longer a gene is, the higher the amount of fragments that come from it. In addition, the longer the gene is, the more likely it is that one of the reads aligns against this gene. As we are concerned with analyzing not only differential expression between normal and tumor cells, but also with differential expression among genes of the same family, raw gene data was normalized for the corresponding gene length (*l*) and the size of the library samples (*n*), thus making expression values among different genes readily comparable. As expression values after normalization are very small, they were multiplied by 10^9^ to obtain expression values as Reads per Kilobase per Millions of mapped reads (RPKM) that can be obtained using the following expression (1).

(1) $$\mathrm{RPKM}=\frac{\mathrm{reads}}{l\cdot n}\cdot 10^{9}$$

**Figure 1:**
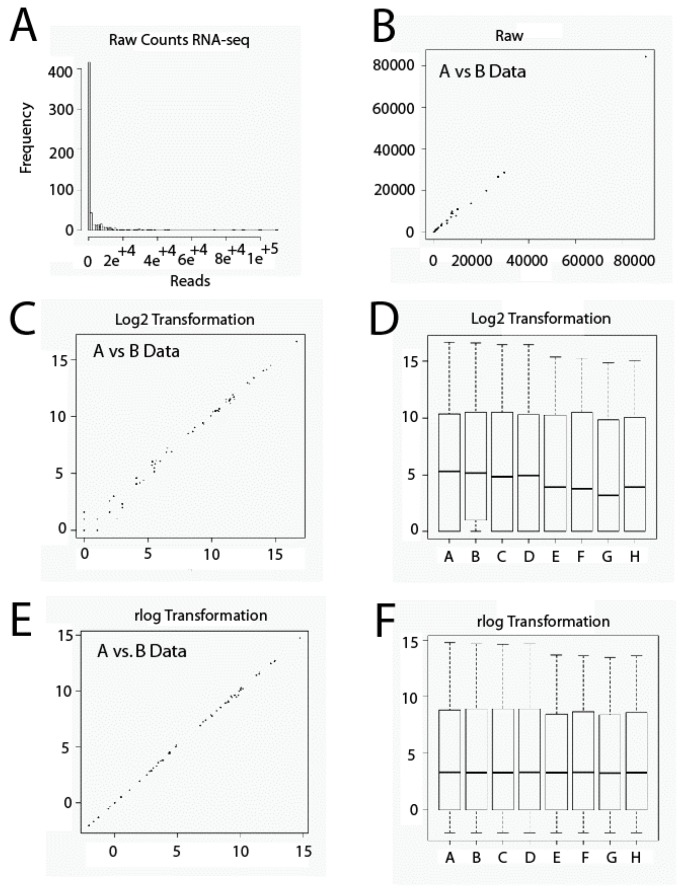
Gene expression level data transformation. Raw data set values (**A**,**B**). Log2 transformation (**C**,**D**); rlog transformation (**E**,**F**). RNA-seq: RNA-sequencing.

Another important issue relative to RNA-seq data sets is that they contain usually a large amount of low and disparate values. Thus, it is very important to be careful with the scale employed. If we use a raw data set, almost all data will plot on low values and only a few on higher values, thus occluding large parts of data (Figure 1A,B). This problem could affect techniques of Exploratory Data Analysis such as Principal Components Analysis or Cluster Hierarchical Analysis, since highly expressed genes could dominate them. To solve this issue, raw data has to be transformed numerically using, for example, Box-Cox Transformations or Logarithmic Transformations. In the present study, we used a data transformation similar to logarithmic transformation in base 2. Transformation shrinks data variation across samples. However, low-expression genes tend to over-dominate when this transformation is used. This is due, on one hand, to Poisson noise, related to low values of reads and, on the other hand, to the fact that logarithm transformation amplifies differences among low values. Thus, low-expression genes will show relatively larger differences among samples (Figure 1C,D). To solve this problem, we have used Regularized Logarithm Transformation (rlog). In this case, the outcomes of highly expressed genes are similar to outcomes obtained by logarithmic transformation in base two. In addition, differences among low values essentially disappear (Figure 1E,F). 

**Figure 2:**
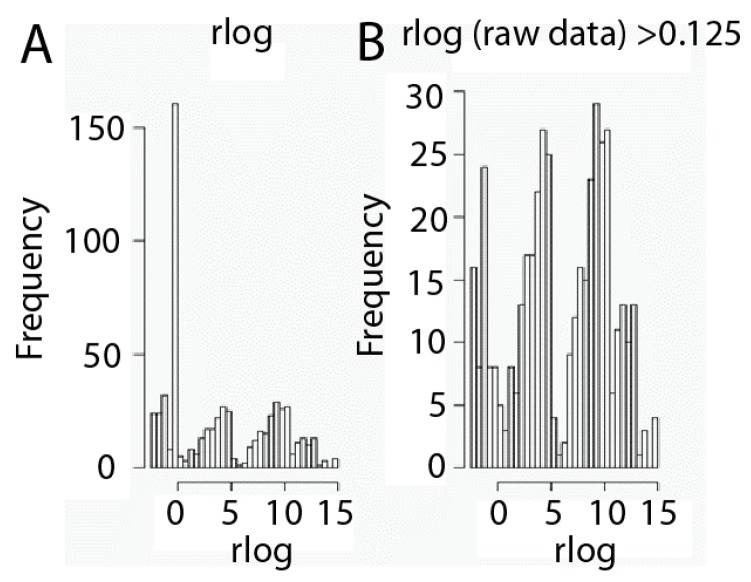
Gene expression level filtering. Histograms of data before (**A**) and after (**B**) the data set filtration process. Filtering was carried out by removing genes with a profile expression lower than 0.125 Reads per Kilobase per Millions of mapped reads (RPKM). Data are shown as rlog transformed.

Another key point of RNA-seq data analysis is filtering in order to minimize hypothesis testing. We have to take into account that RNA-seq data sets contain a large amount of values equal to zero, since not all genes are expressed by every kind of cell, and that is very important relative to filtering. Thus, those genes that may not play any role or show no relationship to phenotype may be removed from the data expression set. Nevertheless, we need to be careful in this step to avoid removing genes which marginally do not show any kind of activity in a cell but may act jointly with other genes. Thus, we have considered taking into account the criteria set up by Hackett [27] and Seyednasrollah [28], who suggested filtering out those genes that are not readily expressed in any of the samples and whose median expression profile is lower than 0.125 RPKM [29]. Data transformed in this manner were filtered in order to remove those genes considered as non-expressed, whose median expression profile is lower than 0.125 RPKM (Figure 2). Interestingly, three types of gene populations according to expression values emerge after filtering: genes expressed at low, intermediate and high expression levels (Figure 2).

### 2.2. Exploratory Data Analysis: Hierarchical Clustering

After data transformation and filtration, two different exploratory data analyses were carried out; although they are not hypothesis testing, they are useful for generating hypotheses. The first one, known as Hierarchical Cluster Analysis, is carried out both in samples and genes. This analysis, using Euclidean distance as dissimilarity measurement, allows the breaking up of samples or genes into groups of members sharing common characteristics. It also establishes which genes are co-regulated. The second exploratory data analysis is known as Principal Components Analysis, which is very useful in order to reduce the dimension of the data or to find patterns among genes or samples, thus getting new variables which are a linear combination of the original ones.

Hierarchical Clustering Analysis forms observation clusters whose members share common characteristics. For example, it is possible to detect different populations, such as healthy and tumor phenotypes, thereby clustering each sample into a healthy or tumor group. Furthermore, if the existence of different groups is known, it could be possible to identify a rule to sort the observations. For example, we can sort samples with Discriminant Analysis [30]. In the present study, we clustered both samples and genes. Thus, if we classify samples, we can discover the different populations they came from and, if we classify genes, we can use the information to carry out, for example, an Enrichment Gene Set Analysis that identifies which gene clusters are related to the tumoral phenotype. However, this analysis was not undertaken here because of the low number of genes studied and for the sake of brevity.

**Figure 3:**
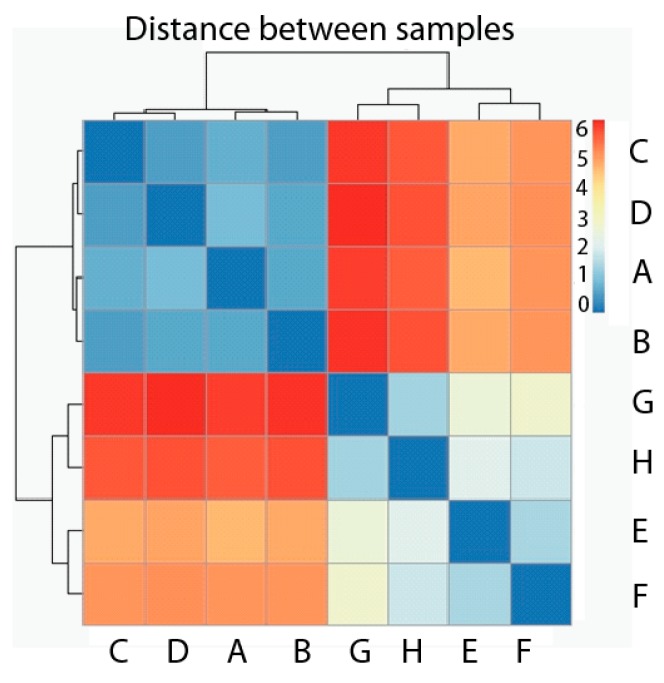
Heatmap Hierarchical Cluster of samples with healthy phenotype (**A**–**D**) and tumor phenotype (**E**–**H**). Pseudocolor scale shows hierarchical distance from minimum (0, blue) to maximum (6, red).

We first carried out the Hierarchical Cluster Analysis considering the eight samples used as observations and genes as variables. The dissimilarity measurement chosen is Euclidean distance, and the analysis is shown as a heatmap with dendograms in both axes of Figure 3. According to the map, it is clear that two groups have been formed, where the first one contains all four samples belonging to the normal, healthy phenotype (A, B, C, D), and the second one contains all four samples corresponding to the tumor phenotype (E, F ,G ,H) (Figure 3). The distance among samples belonging to the same phenotype is much smaller than the distance among samples belonging to different phenotypes. Each value represented in the heatmap corresponds with the distance between each sample couple. 

**Figure 4:**
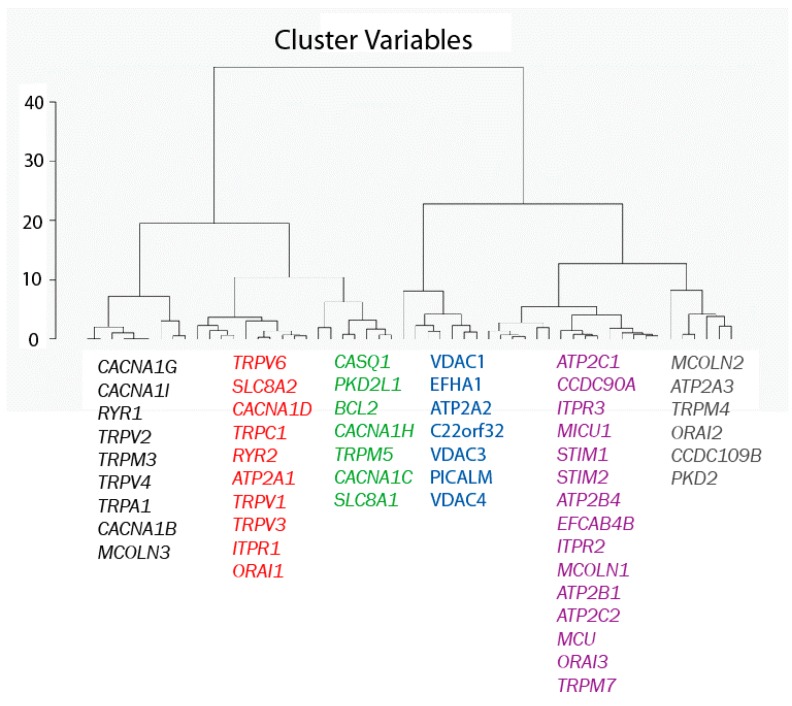
Hierarchical clustering of genes. Genes have been divided into six different groups according to their Euclidean distances relative to their expression values (in RPKM).

A second Hierarchical Clustering Analysis was carried out in a similar way, except that now the 77 genes are considered as observations and the eight samples as variables. The result of the analysis is shown in Figure 4. The Euclidian distances map shows that the 77 genes in the pool break into six different groups. All the genes in each group behave similarly in the sense that, regardless of whether they are high or low expressed in normal and tumor cells, expression levels are similar for all genes within each group and different from expression levels of the other gene families according to the Euclidian distances shown in Figure 4. 

### 2.3. Gene Correlations

**Figure 5:**
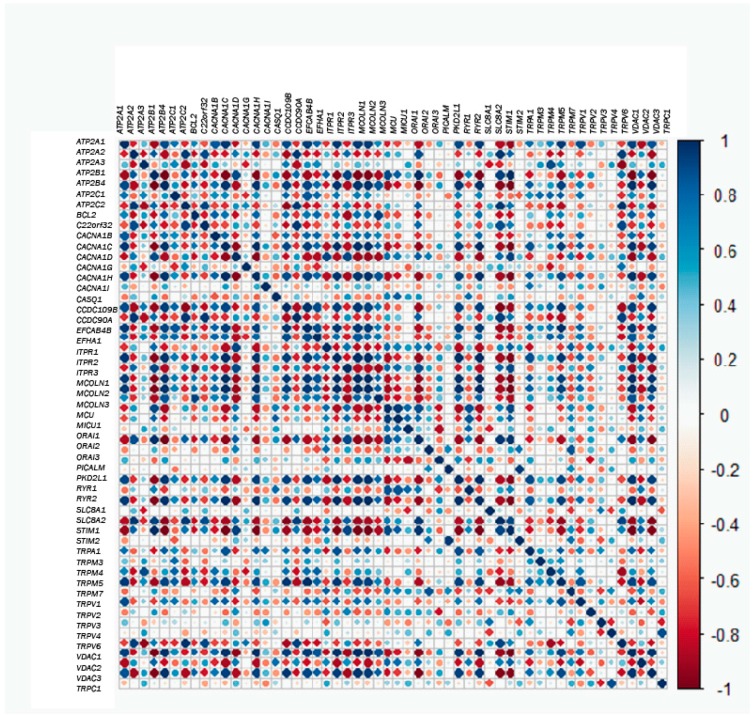
Correlation or co-regulation between couples of genes. The larger the circle and the darker the color, the higher the correlation (either positive in blue or negative in red) between each pair of genes.

Next, we questioned possible correlations between gene couples. In this way, correlation values for each gene couple were plotted in the heatmap shown in Figure 5. Thus, in the case that two genes are positively and strongly correlated, if the expression of one of them is low in tumor samples, the expression of the other one will also be low in the normal sample. Conversely, if the pair of genes is negatively and strongly correlated and the expression of one of them is low in tumor samples, the expression of the other one would be very high in normal cells (Figure 5). Thus, for correlated genes, expression of one of them will allow prediction of the behavior of the correlated genes. For instance, *MCU* and *MICU1* are very positively co-regulated with *Orai2* and *RYR1* and are thus extremely and negatively co-regulated with *RYR2*. Thus, when the expression of *MCU* and *MICU1* increases, the expression of *Orai2* and *RYR1* will be also high, whereas the expression of *RYR2* would be much lower. Nevertheless, not all genes are co-regulated, either positively or negatively, and there are many genes that are not co-regulated. For example, *MCU* and *MICU1* do not co-regulate with *ATP2A3*, and they are almost uncorrelated with *TRPM3* and *TRPM4*. Therefore, while the expression of *MICU1* and *MCU* is enhanced in the tumor phenotype, the expression of *ATP2A3*, *TRPM3* and *TRPM4* seems not to vary much. Therefore, this analysis enabled us to uncover which genes behave in the same way and which do not when comparing healthy and tumor phenotypes. In addition, it can predict whether or not the behaviors are similar.

### 2.4. Principal Component Analysis

**Figure 6:**
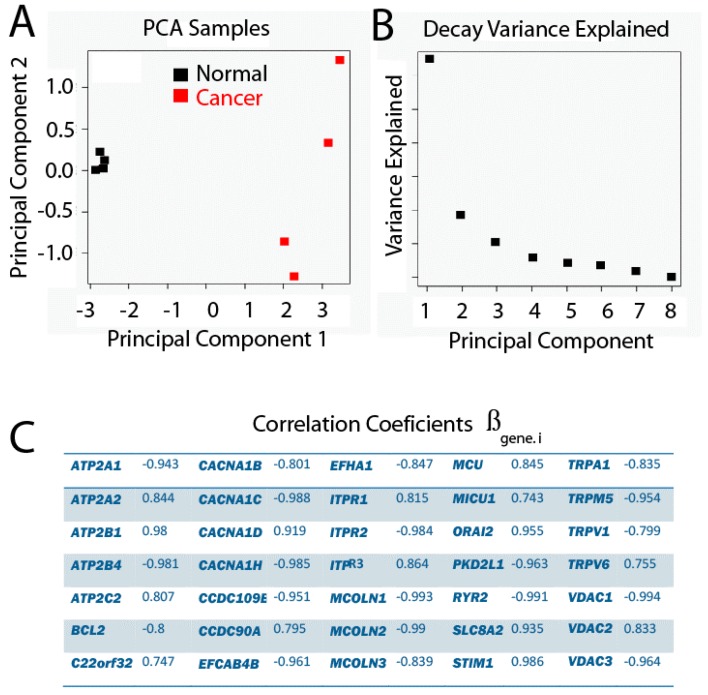
Principal Component Analysis (PCA), where genes are variables and samples are observations. The proportion of variance explained by PC1 is equal to 59.82%, and 16.19% for PC2. (**A**) PC2 vs. PC1; (**B**) Decay Variance Explained graph; (**C**) Correlation coefficients.

The Principal Component Analysis describes the variation produced by a multivariate observation, such that new variables are made from linear combinations of the original variables. These new variables are known as Principal Components (PCs). Thus, if the observation has p original variables, up to p PCs can be made, which are sorted by the amount of explained variance by each of them, where PC1 explains the largest amount of variance, followed by PC2, and so on. Therefore, this analysis is intended to reduce the dimension of the observations. With the new dimensions selected, PCs explain the largest possible amount of variance. Indeed, a criterion for deciding how many PCs to keep is that the proportion of variance explained for all PCs selected is larger than 70%. Other criteria are taken from the Decay graph, which represents the explained variance by each PC against the corresponding PC. Thus, the number of PCs located in the Decay graph before the slope of the graph changes drastically (Figure 6) shows that the variance explained does not increase much despite considering more PCs. 

As it is really difficult for multivariate data to verify the assumption that they fit a normal distribution, the Principal Component Analysis is considered as a kind of exploratory data analysis. This is why data have been filtered and transformed previously—to fit normal distribution as much as possible. Furthermore, data have been centered with their mean, and standardized with their variance. 

In the present study, a Principal Component Analysis between samples as a function of the expression profile of p genes has been carried out and the results are shown in Figure 6. We found that PC1 clearly explains the difference between phenotypes, since the projections of the values for each sample over PC1 show how the samples belong to a healthy phenotype and are well separated from the samples belonging to the tumor phenotype. Since the variance explained by PC1 is 59.82%, and the one explained by the two first PCs is 76.79%, together with the fact that the slope of the Decay graph changes drastically from PC2, it is a good decision to keep only the first two PCs. 

Given that PC1 clearly reproduces the two different groups related with the phenotype, it is interesting to evaluate the influence of each original variable gene, which is proportional to a coefficient associated with the linear combination for each gene. The way in which this is evaluated in the present study is by estimating the correlation between each gene and PC1. Genes with correlations larger than |0.7| are shown in Table 2. 

Accordingly, it is possible to obtain the following expression (2):

(2) $$\mathrm{PC}1=\overset{p}{\underset{i\;=\;1}{\sum }}rlog\left(expression\;of\;gene.i\right)\cdot \beta_{gene.i}$$

where PC1 is the value of the Principal Component 1, *rlog*(*expression of gene.i*) is the value of expression for each gene after transformation (see supplementary data for raw expression data of individual genes) and *βgene.i* is the coefficient value for the same gene (Table 2). This enables the sorting of a given sample to the normal or tumor phenotype. It is important to take into account that the values of the original variables have been transformed, centered and standardized as reported.

### 2.5. Differential Expression Analysis

There is no consensus on the best way to carry out a Differential Expression Analysis. It is also debatable which methods perform best as well as which probability distribution fits best to a given data set [28,31]. In the present study, a data set shows a low sequencing depth, unbalanced sequencing depth among samples, and low number of technical replicates (*n* = 4). In order to keep a relatively conservative approach and, at the same time, identify a good number of genes differentially expressed, three different methods were used: EdgeR [32], DESeq2 [33] and Limma [34]. EdgeR is the less conservative method because it detects the largest number of true positives (genes which are indeed expressed differentially) at the expense of introducing more false positives. In contrast, DESeq2 is considered the most conservative one [31]. Thus, Zhang et al. [31] recommend using EdgeR when the experimental design is unbalanced and sequencing depth and number of replicates are low. However, Seyednasrollah [28] suggests using Limma because of its good documentation and speed, as well as ease of use and robustness. Thus, we followed the suggestion by Zhang et al. [31] of taking the genes differentially expressed which belong to the intersection from these three methods in order to avoid a high amount of false positives. Accordingly, we used an R/Bioconductor package called Enrichment Browser [35], and applied Limma, EdgeR and DESeq2 methods of differential expression analysis. Only those genes whose differential expression was considered significant by all three independent methods were considered to be expressed differentially. Table 3 shows fold changes for all 30 considered differentially expressed genes out of the 77 analyzed, along with the three *p* values obtained by the three independent methods. We found that 12 genes were significantly upregulated in CRC cells (positive fold change) while the remaining 18 genes were significantly downregulated (negative fold change). The most robust changes corresponded to the transient receptor potential channel, vaniloid 6 (*TRPV6*) and the sodium-calcium exchanger 2 (*NCX2*) for upregulated genes in CRC, and ryanodine receptor 2 (*RyR2*) and voltage-dependent Ca^2+^ channel 1.2 (*Cav1.2*) for downregulated genes. In order to appreciate best the differential gene expression, changes corresponding to each family of genes are detailed next. 

**Figure 7:**
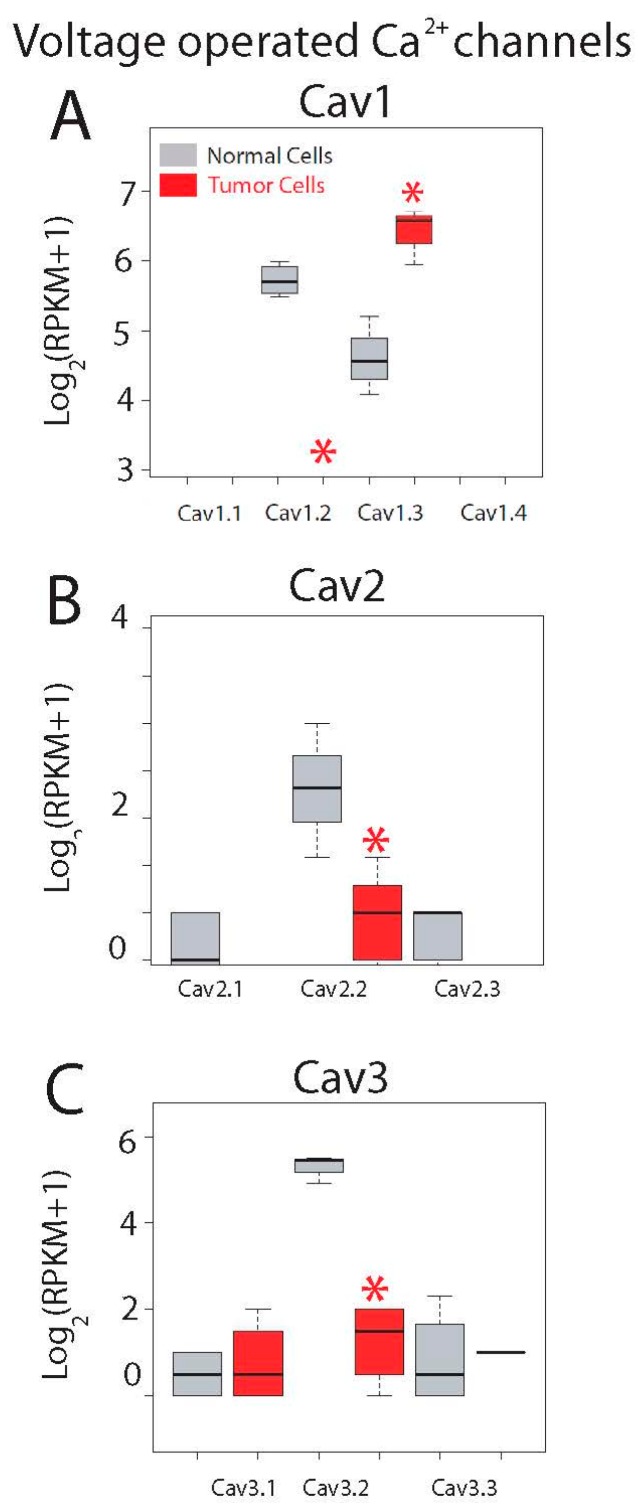
Expression of voltage-gated Ca^2+^ channels in normal and colon cancer cells. Expression levels of Cav1s (**A**), Cav2s (**B**) and Cav3s (**C**) in normal colonic cells (grey bars) and colon cancer cells (red bars). * *p* statistically significant by three independent methods.

Expression levels for all ten voltage-operated Ca^2+^ channels (VOCCs) are shown in Figure 7. This family is formed by three subfamilies named Cav1, Cav2 and Cav3. Although epithelial colonic cells are not electrically excitable cells and lack functional voltage-operated Ca^2+^ entry, both normal and colon cancer cells express mRNAs for some VOCCs. Cav1.1 to Cav1.4 are L-type VOCCs sensitive to dihydropyridines. Only Cav1.3 is expressed in both normal colonic and colon cancer cells and expression levels are significantly enhanced in tumor cells (Figure 7A). Cav1.2 is expressed only in normal cells and the remaining Cav1.1 and Cav1.4 are missing in both cell types (Figure 7A). 

The second subfamily of VOCCs, Cav2s, include P/Q and R type channels typically restricted to neurons. Only Cav2.2 is expressed in normal colonic cells and expression is essentially lost in CRC cells (Figure 7B). 

Finally, Cav3.1 to Cav3.3 are T-type Ca^2+^ channels activated only at high potentials and expressed typically in cardiac and smooth muscles as well as in some neurons. Only Cav3.2 is expressed in normal colonic cells and expression is also nearly lost in CRC cells (Figure 7C). Interestingly, it has been shown recently that blockers of T-type channels may prevent colon cancer growth [36]. Thus, transcripts of Cav1.2, Ca1.3, Cav2.2 and Ca3.2 are differentially expressed in normal colonic and CRC cells. In any case, we must take into account that expression of these channels at the protein level may or may not correlate with expression at the messenger level. 

**Figure 8:**
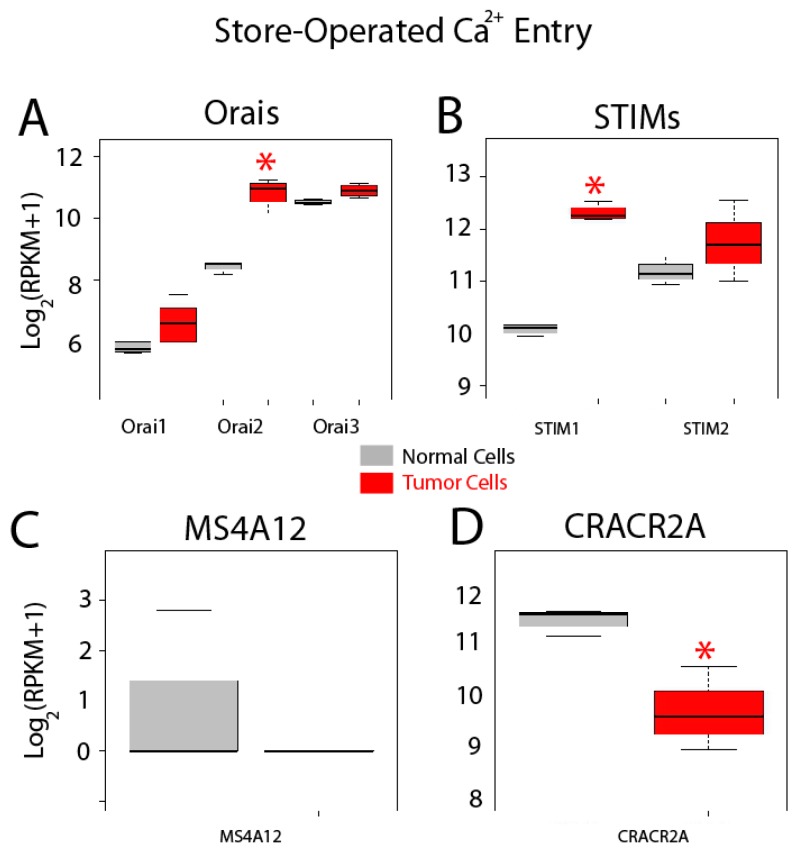
Expression of genes involved in store-operated Ca^2+^ entry in normal and colon cancer cells. Expression levels of *Orais* (**A**), *STIMs* (**B**), *MSA412* (**C**) and CRACR2A (**D**) in normal colonic cells (grey bars) and colon cancer cells (red bars). * *p* statistically significant by three independent methods.

Gene expression levels for molecular players involved in SOCE are shown in Figure 8. Store-operated Ca^2+^ entry, also named capacitative Ca^2+^ entry, is activated after agonist-induced emptying of intracellular Ca^2+^ stores. This pathway is mediated by interactions between STIM sensors at the ER and Orai channels at the plasma membrane. All three *Orais* are expressed in both normal and colon cancer cells and expression appears to be increased for all of them (Figure 8A). However, only differences for *Orai2* mRNA are significant in tumor cells (Figure 8A). *STIM* and 2 are also expressed in normal and colon cancer cells (Figure 8B). Interestingly, *STIM1* is significantly enhanced in tumor cells, whereas *STIM2* remains similar (Figure 8). In addition to these players, *MS4A12* has been reported to be a colon-selective store-operated Ca^2+^ channel promoting malignant cell processes [37]. However, MS4A12 mRNA is barely or not expressed either in normal cells and CRC cells (Figure 8C). Finally, CRACR2A, a SOCE regulatory protein [38], is expressed in both normal and tumor cells and expression levels are diminished in tumor cells (Figure 8D). 

**Figure 9:**
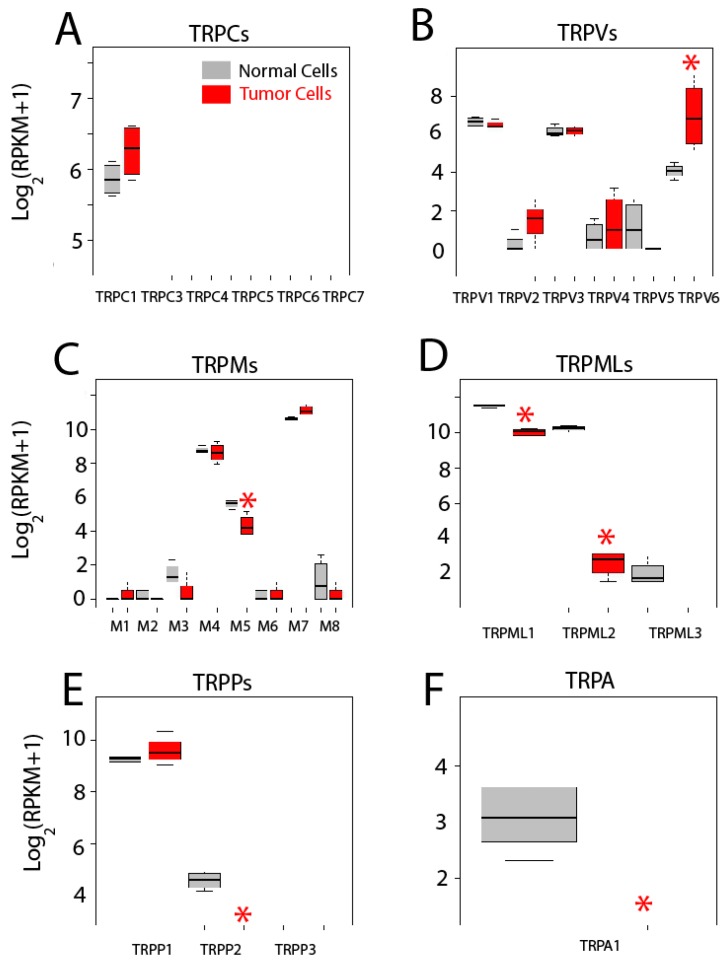
Expression of transient receptor potential (TRP) channels in normal and colon cancer cells. Expression levels of canonical transient receptor potential channels *TRPCs* (**A**), canonical Transient Receptor Potential channels *(TRPVs*) (**B**), Melastatin Transient Receptor Potential channels (*TRPMs*) (**C**), Mucolipin Transient Receptor Potential channels (*TRPMLs*) (**D**), Polycystin Transient Receptor Potential channels (*TRPPs*) (**E**) and Transient Receptor Potential channel member A, subfamily 1 *(TRPA1*) (**F**) in normal colonic cells (grey bars) and colon cancer cells (red bars). * *p* statistically significant by three independent methods.

We have assessed differential gene expression of all 27 human TRP channels (Figure 9). TRP channels are cationic channels that may permeate Ca^2+^ with variable selectivity and are involved in disparate cell functions, some of them contributing to SOCE, particularly the canonical TRP channels (TRPCs). There are seven members of the TRPC family of cation channels TRPC1 to TRPC7. RNA-seq data shows that both colonic and CRC cells express only *TRPC1* and expression seems increased in colon cancer cells. However, difference is not significant (Figure 9A). These results are at variance with our previous results using quantitative reverse transcription-polymerase chain reaction (qRT-PCR) [4]. Differences could be explained by increased sensitivity and/or threshold for significance in our present analysis. In any case, we have confirmed that expression of TRPC1 protein is significantly enhanced in CRC [4], stressing further the need for protein and functional confirmation of transcriptomic/genetic analysis. 

The vaniloid family of transient receptor potential channels (TRPVs) are a very heterogeneous family of six cationic channels from TRPV1 to TRPV6. Only *TRPV1*, *TRPV3* and *TRPV6* are expressed in colonic cells. Interestingly, while expression levels of *TRPV1* and *TRPV3* are similar in normal and CRC cells, *TRPV6* is significantly enhanced in tumor cells (Figure 9B). This channel is involved in vitamin D-dependent absorption of extracellular Ca^2+^ in the intestines. Interestingly, it has been previously reported that TRPV6 is overexpressed in prostate cancer [2,3]. 

The melastatin family of transient receptor potential channels (TRPMs) are a big family of eight cation channels from TRPM1 to TRPM8. Only three of them, *TRPM4*, *TRPM5* and *TRPM7*, appear to be expressed in colonic cells. Levels of *TRPM4* and *TRPM7* are similar in normal colonic and CRC cells. However, expression of *TRPM5* decreases significantly in CRC cells (Figure 9C).

The mucolipin family of transient receptor potential channel (TRPMLs) are a family of three channels, TRPML1 to 3, also named mucolipins. Only the two first mucolipines are expressed in normal and tumor cells and both are significantly decreased in CRC cells. Particularly noteworthy is the case of TRPML2 that is dramatically downregulated in colon cancer cells (Figure 9D). 

The polycystine family of transient receptor potential channels (TRPPs) are also a family of three channels, TRPP1 to TRPP3, TRPP1 being the best known for its role in polycystic kidney disease. *TRPP1* is expressed in both normal and colon cancer cells to the same extent (Figure 9E). *TRPP2* is expressed only in normal cells (Figure 9E) and *TRPP3* is absent in both normal and colon cancer cells (Figure 9E). 

Finally, the transient receptor potential channel, member A, subfamily 1 (TRPA1), a stress sensor and only member of the TRPA family of channels, is expressed in normal colonic cells but not in colon cancer cells (Figure 9F). 

In summary, only 11 out of the 27 TRPs are expressed in colonic cells, either normal or tumor. In addition, only six show differential expression. In all the cases but one, TRP channels are downregulated in CRC cells relative to normal cells, TRPV6 being the only TRP channel significantly increased in CRC cells. Functional consequences of changes in expression of TRP channels in CRC remain to be established.

**Figure 10:**
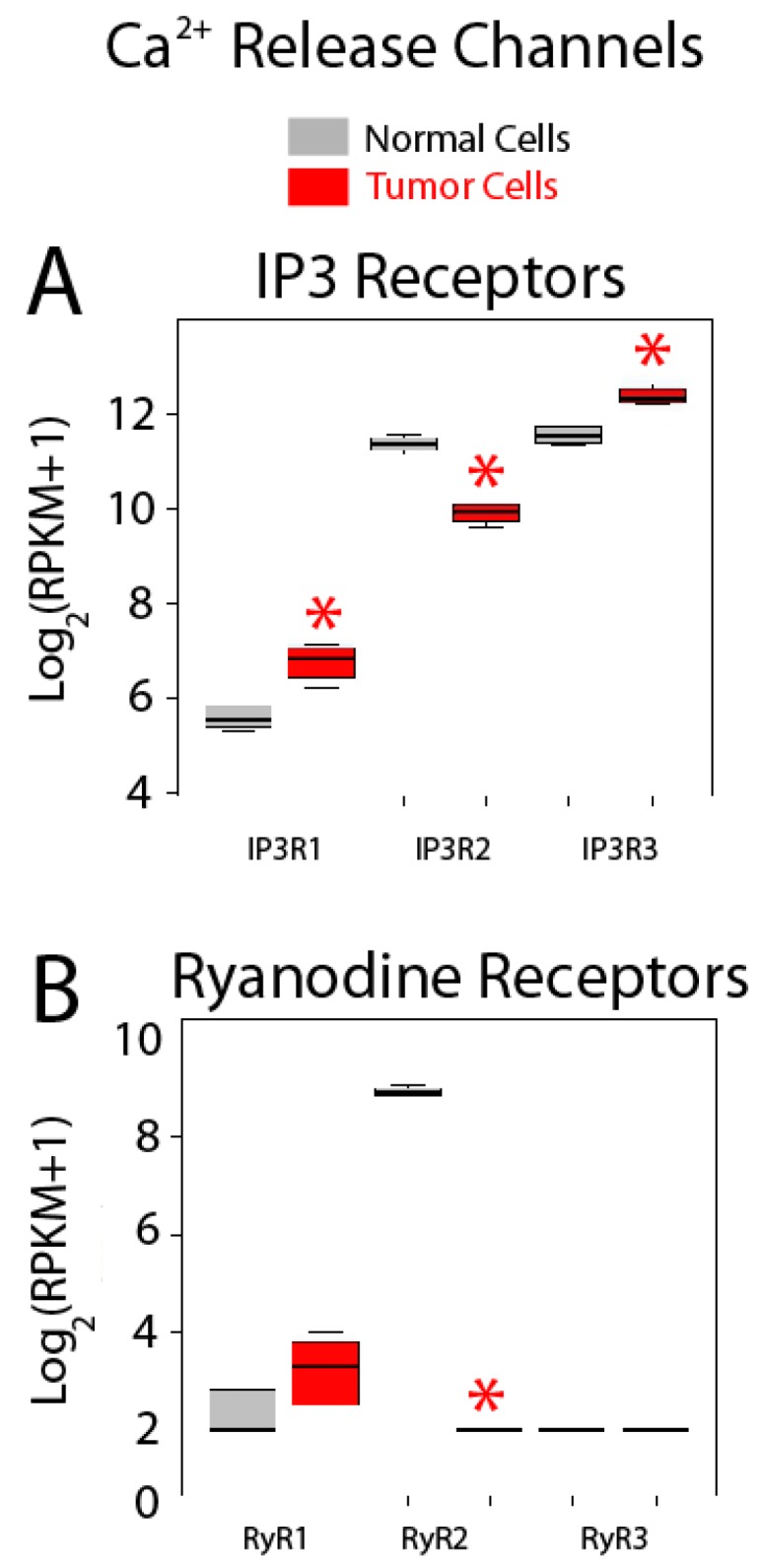
Expression of Ca^2+^ release channels in normal and colon cancer cells. Expression levels of inositol trisphosphate receptors (IP_3_Rs) (**A**) and ryanodine receptors (RyRs) (**B**) in normal colonic cells (grey bars) and colon cancer cells (red bars). * *p* statistically significant by three independent methods.

Expression of Ca^2+^ release channels is shown in Figure 10. IP_3_ and ryanodine receptors are Ca^2+^ release channels at the ER, the most important intracellular Ca^2+^ store. There are three IP_3_ receptor (IP_3_R) subtypes, IP_3_R1 to IP_3_R3, and all of them are expressed in both normal colonic and colon cancer cells (Figure 10A). Interestingly, all three isoforms are also differentially expressed in colon cancer cells relative to normal cells. While genes corresponding to IP_3_R1 and IP_3_R3 are overexpressed in colon cancer cells, gene coding for IP_3_R2 is downregulated (Figure 10A). Interestingly, consistent with these results, it has been reported that the type III IP_3_R is absent in normal colonic cells but is expressed in colon cancer. Moreover, expression levels are directly related to aggressiveness of the tumor [39]. In addition, it has been also reported that depletion of mutated K-Ras in a colon cancer cell line changes Ca^2+^ store content and susceptibility to apoptosis by promoting changes in expression of IP_3_R isoforms [40]. Therefore, IP_3_Rs are critical players in Ca^2+^ remodeling in colon cancer, contributing likely to changes in Ca^2+^ store content and susceptibility to apoptosis. 

Regarding RyRs, only genes coding for RyR1 and 2 are expressed in colonic cells, although RyR2 is by far the most abundant isoform (Figure 10B). In addition, RyR2 is dramatically downregulated in colon cancer cells (Figure 10B). Together, these data indicate that Ca^2+^ release could be substantially influenced in CRC cells relative to normal cells as reported recently [4]. 

**Figure 11:**
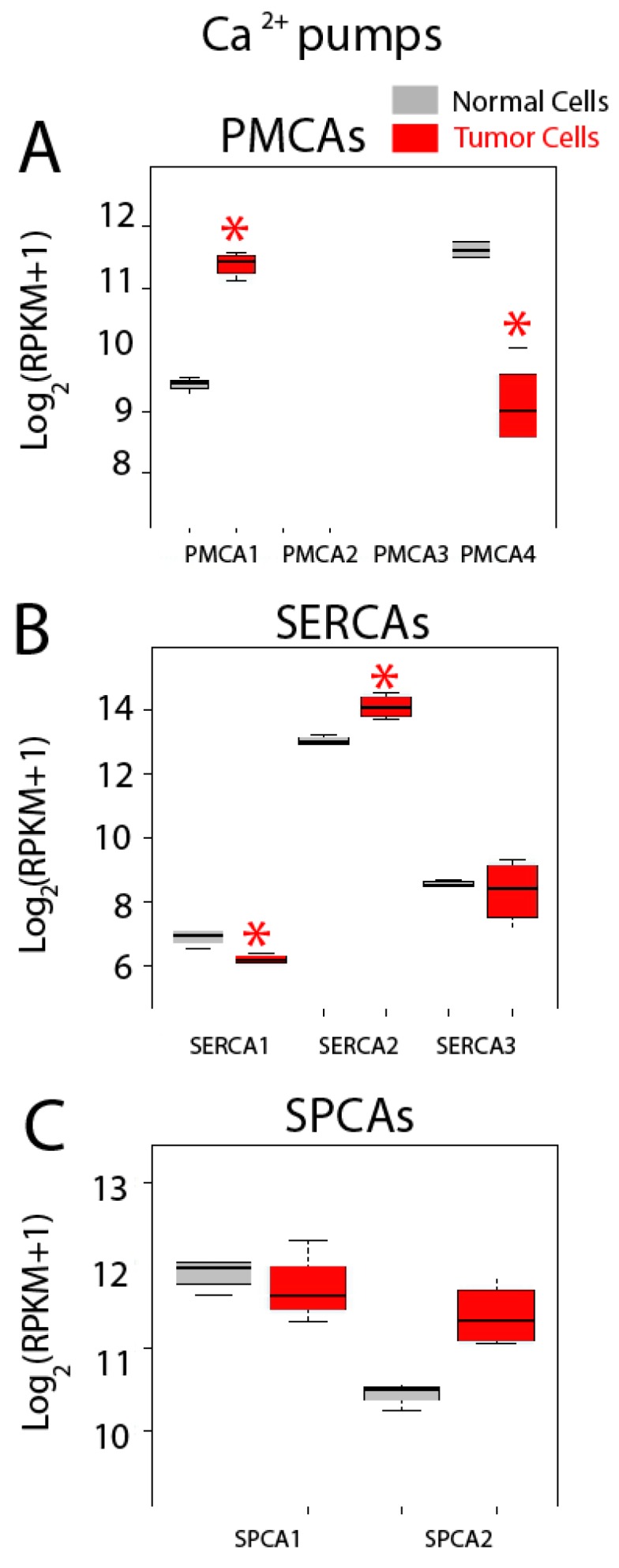
Expression of Ca^2+^ pumps in normal and colon cancer cells. Expression levels of plasma membrane Ca^2+^ ATPases (PMCAs) (**A**), sarcoplasmic and/or endoplasmic reticulum Ca^2+^ ATPases (SERCAs) (**B**) and secretory pathway Ca^2+^ ATPases (SPCAs) (**C**) in normal colonic cells (grey bars) and colon cancer cells (red bars). * *p* statistically significant by three independent methods.

Gene expression levels for Ca^2+^ pumps including PMCAs, SERCAs and SPCAs are shown in Figure 11. Regarding genes coding for PMCAs, we found that only PMCA1 and PMCA4 are expressed in both normal and colon cancer cells, while PMCA2 and PMCA3 are absent in both cell types (Figure 11A). Interestingly, PMCA1 expression increases in cancer cells while expression of PMCA4 is downregulated in tumor cells (Figure 11A). Thus, data suggest a molecular switch from PMCA4 to PMCA1 in CRC. These results are consistent with a previous report showing that undifferentiated colonic cell lines express PMCA1 and differentiation downregulates it while promoting expression of PMCA4 [41]. 

All three SERCAs are also expressed in both normal and tumor cells (Figure 11B). Interestingly, SERCA1 expression decreases significantly in tumor cells while SERCA2 is upregulated in tumor cells. No difference is observed in SERCA3 (Figure 11B). Thus, a molecular switch from SERCA1 to SERCA2 may be also involved in CRC. Finally, both SPCAs are expressed in normal and colon cancer cells but expression levels are similar (Figure 11C). Although SPCA2 seems to be overexpressed in cancer cells, differences were significant by only two of the three methods used to assess significance. Changes in SERCA activity could lead to possible differences in Ca^2+^ store content between normal and CRC cells. In fact, we have reported recently that CRC cells display the partial depletion of Ca^2+^ stores [4]. This effect has been partially attributed to loss of ER Ca^2+^ sensor STIM2 [4]. Whether changes in SERCA isoform contribute to this remodeling as well remains to be established. 

**Figure 12:**
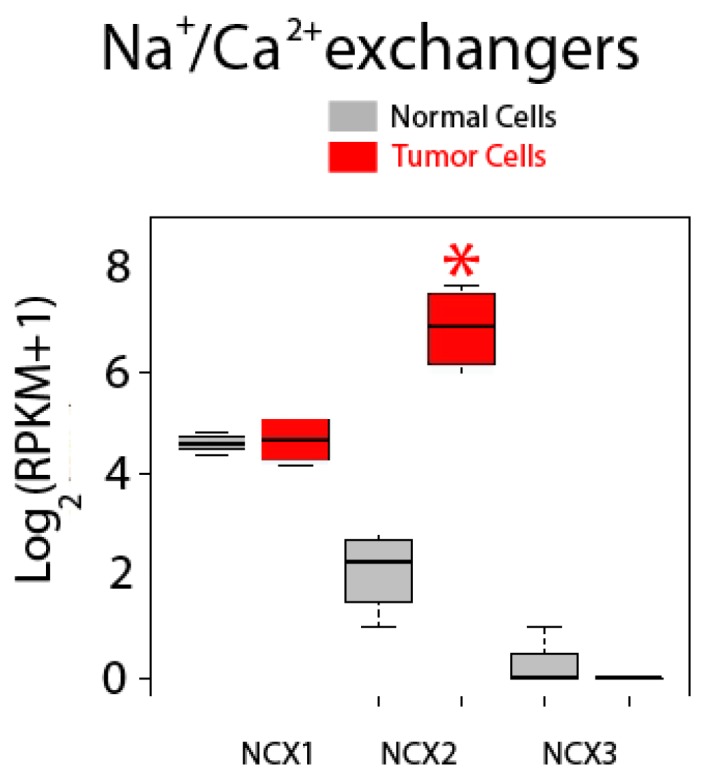
Expression of Na^+^/Ca^2+^ exchanger isoforms in normal and colon cancer cells. Expression levels of NCXs in normal colonic cells (grey bars) and colon cancer cells (red bars). * *p* statistically significant by three independent methods.

Expression of Na^+^/Ca^2+^ exchanger isoforms is shown in Figure 12. These co-transporters normally extrude Ca^2+^ back to the external medium in exchange for Na^+^ using the electrochemically favorable gradient for Na^+^. There are three isoforms named NCX1, NCX2 and NCX3, but only NCX1 and NCX2 are expressed in normal and colon cancer cells. Interestingly, while expression of NCX1 is similar in normal and CRC cells, expression of NCX2 is dramatically enhanced in CRC cells, actually showing the largest fold increase in expression in tumor cells of all upregulated genes tested (Figure 12). 

**Figure 13:**
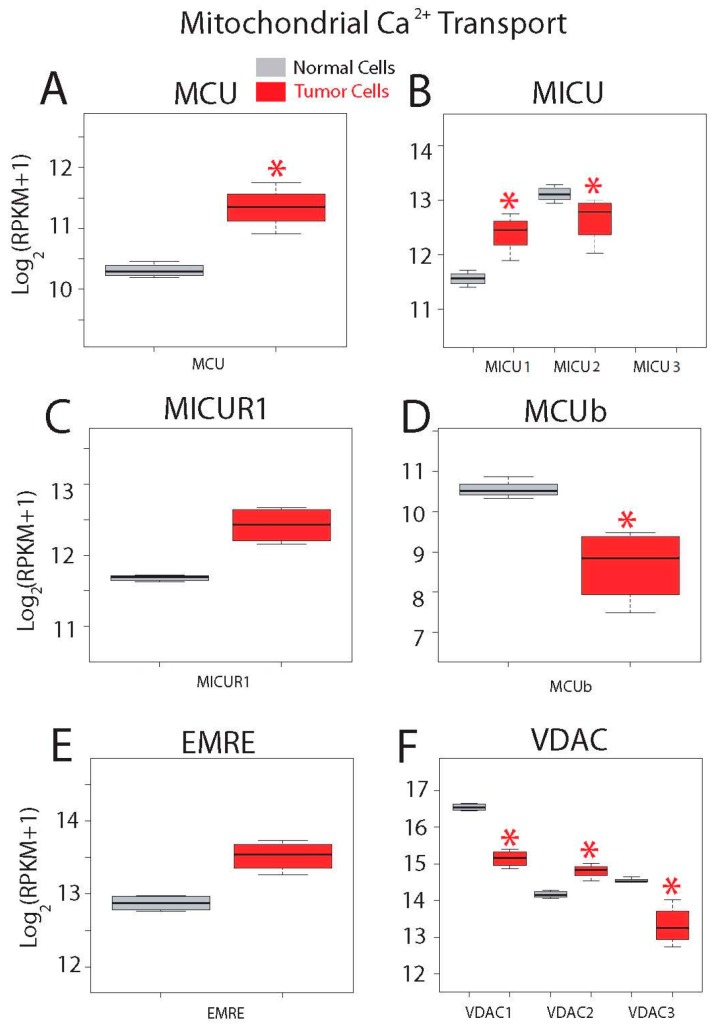
Expression of genes involved in mitochondrial Ca^2+^ transport in normal and colon cancer cells. Expression levels of Mitochondrial Calcium Uniporter (MCU) (**A**), Mitochondrial Calcium Uptake (MICU) isoforms (**B**), Mitochondrial Calcium Uptake Regulator 1 (MICUR1) (**C**), Mitochondrial Calcium Uniporter Dominant Negative Beta Subunit (MCUb) (**D**), Essential MCU regulator (EMRE) (**E**) and voltage-dependent anion channels (VDACs) (**F**) in normal colonic cells (grey bars) and colon cancer cells (red bars). * *p* statistically significant by three independent methods.

Gene expression levels for molecular players involved in mitochondrial Ca^2+^ transport are shown in Figure 13. Ca^2+^ enters mitochondria down its electrochemical gradient through the mitochondrial Ca^2+^ uniporter (MCU) that is modulated by several regulatory proteins including mitochondrial Ca^2+^ uptake (MICU) isoforms 1 to 3, mitochondrial calcium uniporter regulator 1 (MICUR1), mitochondrial calcium uniporter dominant negative beta (MCUb) and MCU regulator (EMRE). Voltage-dependent anion channels (VDAC) 1 to 3 in the outer mitochondrial membrane have been involved in apoptosis. Interestingly, messengers involved in mitochondrial Ca^2+^ transport are very highly expressed in both normal and CRC cells (Figure 13). In addition, all genes but MICU3 are expressed in both normal and tumor cells (Figure 13). Expression of the channel MCU and its positive modulator MICU1 is enhanced in tumor cells (Figure 13A,B), while expression of negative modulator MICU2 is decreased. Expression of other MCU modulators including MICUR1 and EMRE is similar (Figure 13B,C,E), while MCUb is significantly downregulated in colon cancer cells (Figure 13D). Finally, all three VDACs are expressed at very high levels in both normal and tumor cells (Figure 13F). Interestingly, expression of all of them is significantly different in colon cancer cells. VDAC1 and 3 are downregulated in colon cancer cells while VDAC2 is significantly overexpressed in cancer cells. 

**Figure 14:**
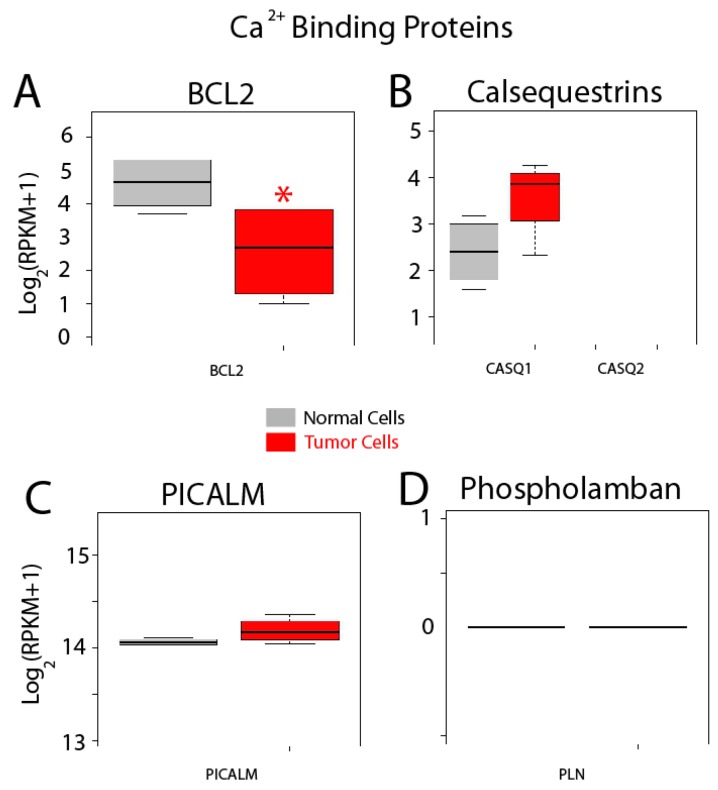
Expression of selected genes coding for other selected proteins. Expression levels of *BCL2*, (**A**) calsequestrins *CASQ1* and *CASQ2* (**B**), *PICALM* (**C**) and *PLN* (Phospholamban) (**D**) in normal colonic cells (grey bars) and colon cancer cells (red bars). * *p* statistically significant by three independent methods.

Finally, expression of other related proteins is shown in Figure 14. Bcl-2 modulates Ca^2+^ release and apoptosis. This gene is significantly downregulated in cancer cells (Figure 14) while expression of two other proteins including calsequestrin 1 and phosphatidylinositol binding clathrin assembly protein (PICALM), is similar (Figure 14B,C). No expression of calsequestrin2 and phospholamban is observed in normal and colon cancer cells. 

**Figure 15:**
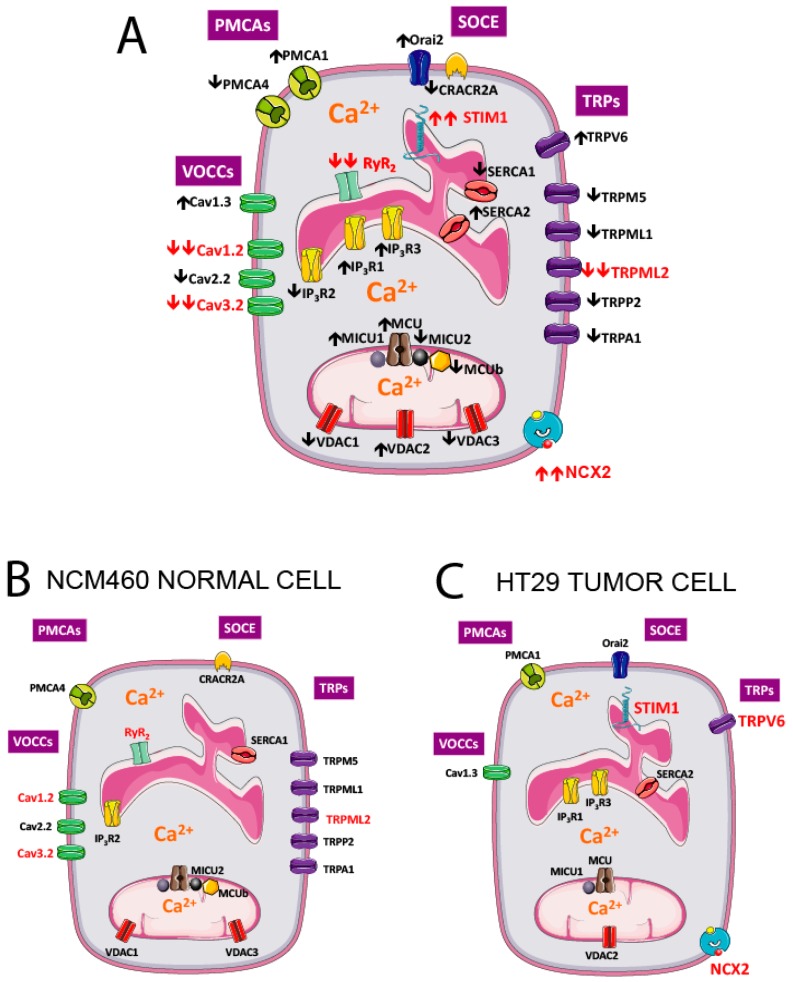
Molecular players differentially expressed in NCM460 normal colonic and HT29 colorectal cancer cells. Genes significantly upregulated (↑) and downregulated (↓) in colorectal cancer cells vs. normal colonic cells are shown in specific locations in the plasma membrane, the endoplasmic reticulum (ER) and mitochondria (**A**). To best appreciate the differences, molecular players downregulated in cancer cells have been removed in the prototypical, normal colonic cell (**B**), whereas only genes upregulated in CRC are shown in the prototypic CRC cell (**C**). Molecular players in red are those with the largest fold changes in expression from the normal to the tumor phenotype. PMCAs, plasma membrane calcium ATPases; VOCCS, voltage-operated calcium channels; SOCE, store-operated calcium entry; TRPs, Transient Receptor Potential channels.

Changes in expression of all significantly expressed transcripts are shown in Figure 15A. To best appreciate the differences, all genes that are significantly downregulated in tumor cells are removed for the genes shown in normal cells (Figure 15B) but show up in the tumor cell (Figure 15C). 

In summary, we report here a comprehensive transcriptomic analysis of Ca^2+^ remodeling in colorectal cancer cells. A similar analysis has been recently carried out in glioblastoma using data gathered from repository data sets of a large series of glioblastomas [23]. Data shown here were generated using Ion Torrent RNA-sequencing from mRNA isolated from normal human colonic NCM460 cells and colon cancer HT29 cells. Therefore, while the study has the limitation that both cell lines may not reflect entirely normal human colonic and colon cancer cells, respectively, it does profit from the fact that changes in intracellular Ca^2+^ handling between the two cell lines have been analyzed in detail [4]. The results provide a strong molecular basis for Ca^2+^ remodeling in CRC. 

## 3. Materials and Methods

### 3.1. Materials 

HT29 cells were donated by José Carlos Fernández-Checa (CSIC, Barcelona, Spain). NCM460 cells were obtained after a material transfer agreement with INCELL Corporation (San Antonio, TX, USA). Dulbecco’s Modified Eagle’s Medium (DMEM), penicillin, streptomycin, l-glutamine and fetal bovine serum came from Lonza (Basel, Switzerland). M3:10TM medium is from INCELL Corporation, San Antonio, TX, USA). 

### 3.2. Cell Culture

Cells are cultured in DMEM 1 g/L glucose or in M3:10TM medium as reported previously [4] and supplemented with 1% penicillin-streptomycin, 1% l-glutamine and 10% fetal bovine serum. Cells are maintained in standard conditions (37 °C, 10% CO_2_) and cultured once a week. All cells were used at passages 3 to 10. 

### 3.3. mRNA Isolation and Ion Torrent Reading

Total cellular RNA was isolated from NCM460 and HT29 cells using Trizol reagent (Invitrogen, Carlsbad, CA, USA). Four totally independent samples from each cell line were used for molecular analysis. Extracted RNA integrity was tested by electrophoresis on agarose gels and the purity and concentration were determined by spectrophotometry. RNA was reverse transcribed using a High Capacity cDNA Reverse Transcription Kit (Thermo Fisher Scientific, Waltham, MA, USA) and the cDNA diluted prior to PCR amplification. A targeted, multiplex PCR primer panel was designed using the custom Ion Ampliseq Designer v1.2 (Thermo Fisher Scientific), where target regions were selected for 77 genes directly or indirectly involved in Ca^2+^ transport and listed in Table 1. The panel was designed to amplify PCR products appropriate for use with RNA from eight different cellular cultures, where four of them belonged to one cellular line and the other four to another. Sequencing libraries were prepared with Ion AmpliSeq RNA Library kit (Thermo Fisher Scientific) using the custom primers. After primer digestion, adapters and Ion Xpress Barcode Adapters (Thermo Fisher Scientific) were ligated to the amplicons followed by magnetic bead purification. Amplicon size and DNA concentration were measured using an Agilent High Sensitivity DNA Kit (Agilent Technologies, Santa Clara, CA, USA) according to the manufacturer’s recommendation. A total of eight picomols of each of the eight samples were pooled for emulsion PCR (ePCR) on Ion Sphere Particles (ISPs) using the Ion PGM Hi-Q OT2 Kit (Thermo Fisher Scientific) using the Ion OneTouch 2 Instrument. Following automated Ion OneTouch, enrichment of template-positive ISP samples were loaded on an Ion 316 V2 Chip and sequenced on a Personal Genome Machine (PGM-Thermo Fisher Scientific) System with Ion PGM Hi-Q Sequencing Kit (Thermo Fisher Scientific). Raw sequencing reads were initially filtered for high quality reads, and the adaptors were removed using the Ion Torrent Suite 4.4.3; reads were then aligned with the hg19_rna reference sequence by TMAP [42] using default parameters. Resulting binary format files (BAM) storing sequence data were processed through an in-house quality control (QC) filter in order to keep those reads whose quality is larger or equal than Q20 and remove polyclonal and primer dimers. After that, outcomes show that 253 Mbp were amplified: the length read median is 108 bp. Reads were aligned against hg19_rna reference genome using GATK LeftAlignIndels module, for 77 genes of interest, where average coverage was 93%, and mean raw accuracy was 99.3%. BAM files were aligned. Amplicon primers were trimmed from aligned reads by Torrent Suite 4.4.3. 

### 3.4. Transformation and Filtration of Raw Data Set

Raw data were transformed with an R/Bioconductor package called DESeq2 [33], applying rlog function on data, and normalized against their gene length, obtained from R package EDASeq [43]. The R code used and the raw data employed in this study is provided as a supplementary file data (esetCalcium file) so that anyone can reproduce the results. Subsequently, using R software, the transformed data set was filtered in order to remove those genes that are not expressed by samples; thus genes whose median of expression profile was lower than 0.125 RPKMs were removed [29]. 

### 3.5. Exploratory Data Analysis

A transformed and filtered data set was analyzed through Exploratory Data Analysis. At first, for both samples and genes, a Hierarchical Clustering Analysis using the Euclidean distance was carried out as well as a correlation between different genes, using R software and stats, heatmap [44] and Corrplot [45] R packages. A second method, the Principal Components Analysis, was applied in order to reveal patterns and reduce the dimensionality of the data, using R software and stats, EDASeq [43] and IRanges R packages.

### 3.6. Differential Expression Analysis

In order to carry out a differential expression analysis, three different methods were used to assess significance. These methods are EdgeR [32], DESeq2 [33] and Limma [34], and they were implemented using R software and EnrichmentBrowser R package [35] because this package allows the performing of differential expression analyses either based on Limma, EdgeR or DESeq2, and adjusted *p* value using the Benjamini-Hochberg method [46]. A critical issue is normalization of data. We have used the default method of normalization in each package as suggested by Seyednasrollah [27] which has a low influence on the analysis outcomes. In addition, we have controlled False Discovery Rate (FDR), proposed by Benjamini-Hochberg [46] where the percent of false positives among whole test where null hypothesis has been rejected is controlled. 

## Figures and Tables

**Table 1:** Genes involved in calcium transport analyzed in this study pooled by gene families.

Gene Group	Protein Name (Gene Name)
Voltage Operated Calcium Channels	Cav1.1 (*CACNA1S*); Cav1.2 (*CACNA1C*); Cav1.3 (*CACNA1D*); Cav1.4 (*CACNA1F*); Cav2.1 (*CACNA1A*); Cav2.2 (*CACNA1B*); Cav2.3 (*CACNA1E*); Cav3.1 (*CACNA1G*); Cav3.2 (*CACNA1H*); Cav3.3 (*CACNA1I*)
Store-Operated Calcium Entry Player	Orai1; Orai2; Orai3; STIM1; STIM2; CRACR2A (*EFCAB4B*); MS4A12
TRP Channels	TRPC1; TRPC3; TRPC4; TRPC5; TRPC6; TRPC7; TRPV1; TRPV2; TRPV3; TRPV4; TRPV5; TRPV6; TRPM1; TRPM2; TRPM3; TRPM4; TRPM5; TRPM6; TRPM7; TRPM8; TRPA1; TRPML1 (*MCOLN1*); TRPML2 (*MCOLN2*); TRPML3 (*MCOLN3*); TRPP1 (*PKD2*); TRPP2 (*PKD2L1*); TRPP3 (*PKD2L2*)
Calcium Release Channels	IP_3_R1 (*ITPR1*); IP_3_R3 (*ITPR2*); IP_3_R3 (*ITPR3*); RYR1; RYR2; RYR3
Calcium Pumps	PMCA1 (*ATP2B1*); PMCA2 (*ATP2B2*); PMCA3 (*ATP2B3*); PMCA4 (*ATP2B4*); SERCA1 (*ATP2A1*); SERCA2 (*ATP2A2*); SERCA3 (*ATP2A3*); SPCA1 (*ATP2C1*); SPCA2 (ATP2C2)
Sodium Calcium Exchangers	NCX1 (SLC8A1); NCX2 (SLC8A2); NCX3 (SLC8A3)
Mitochondrial Calcium Transport Proteins	MCU; MICU1; MICU2 (*EFHA1*); MICU3 (*EFHA2*); MCUR1 (*CCDC90A*); EMRE (*C22orf32*); MCUb (*CCDC109B*); VDAC1; VDAC2; VDAC3
Other Proteins	Bcl-2 (*BCL2*); Calsequestrin 1 (*CASQ1*); Calsequestrin 2 (*CASQ2*); PICALM; Phospholamban (*PLN*)

Cav: Ca^2+^ channel; STIM: Stromal Interaction Molecules; TRPC: canonical TRP channels; TRPV: vaniloid family of transient receptor potential channels; TRPM: melastatin family of transient receptor potential channels; TRPP: polycystine family of transient receptor potential channels; IP_3_R: inositol trisphosphate receptors; RYR: ryanodine receptors; PMCA: plasma membrane Ca^2+^ ATPases; SERCA: sarcoplasmic and/or endoplasmic reticulum Ca^2+^ ATPases; SPCA: secretory pathway Ca^2+^ ATPases; NCX: sodium-calcium exchanger; MCU: mitochondrial Ca^2+^ uniporter; EMRE: MCU regulator; VDAC: voltage-dependent anion channels; PICALM: phosphatidylinositol binding clathrin assembly protein.

**Table 2:** Coefficient values from Principal Component (PC1) corresponding to each gene. Raw data were transformed, filtered, centered and standardized. To obtain PC1 value for a given sample, use coefficients in this table in expression 2.

Gene	Expression	Gene	Expression	Gene	Expression	Gene	Expression	Gene	Expression
*ATP2A1*	−0.166	*CACNA1C*	−0.174	*ITPR1*	0.143	*ORAI3*	0.090	*TRPA1*	−0.147
*ATP2A2*	0.148	*CACNA1D*	0.162	*ITPR2*	−0.173	*PICALM*	−0.089	*TRPM3*	−0.070
*ATP2A3*	−0.088	*CACNA1G*	0.072	*ITPR3*	0.152	*PKD2*	0.020	*TRPM4*	−0.113
*ATP2B1*	0.172	*CACNA1H*	−0.173	*MCOLN1*	−0.175	*PKD2L1*	−0.169	*TRPM5*	−0.168
*ATP2B4*	−0.173	*CACNA1I*	−0.084	*MCOLN2*	−0.174	*RYR1*	0.103	*TRPM7*	0.105
*ATP2C1*	−0.121	*CASQ1*	0.093	*MCOLN3*	−0.148	*RYR2*	−0.174	*TRPV1*	−0.141
*ATP2C2*	0.142	*CCDC109B*	−0.167	*MCU*	0.149	*SLC8A1*	−0.010	*TRPV2*	0.096
*BCL2*	−0.141	*CCDC90A*	0.140	*MICU1*	0.131	*SLC8A2*	0.165	*TRPV3*	−0.060
*C22orf32*	0.131	*EFCAB4B*	−0.169	*ORAI1*	0.061	*STIM1*	0.174	*TRPV4*	0.026
*CACNA1B*	−0.141	*EFHA1*	−0.149	*ORAI2*	0.168	*STIM2*	0.057	*TRPV6*	0.133
*VDAC1*	−0.175	*VDAC2*	0.147	*VDAC3*	−0.170	*TRPC1*	0.041		

**Table 3:** Differential gene expression fold change. Fold changes are expressed as 〖log〗_2 (E_Healthy/E_Tumoral) and E means Expression, and *p*-values adjusted by Benjamini and Hochberg (BH) method for each different package use.

		*p*-Value Adjusted by BH Method		
ID	Fold Change	BH.DESeq2	BH.edgeR	BH.limma
*ATP2A1*	1.02810479	9.71 × 10^−8^	0.0027235	0.00160014
*ATP2A2*	0.83492287	0.00142512	0.00029452	0.02416078
*ATP2B1*	1.66289207	1.32 × 10^−29^	1.69 × 10^−13^	1.58 × 10^−5^
*ATP2B4*	2.75628322	5.53 × 10^-18^	7.86 × 10^−22^	3.01 × 10^−6^
*BCL2*	2.34849379	0.00307301	0.00070215	0.00754796
*CACNA1B*	2.19132122	0.00190951	0.00050421	0.00207432
*CACNA1C*	−11.408836	1.05 × 10^−24^	1.40 × 10^−53^	1.24 × 10^−9^
*CACNA1D*	1.55721384	1.97 × 10^−08^	9.80 × 10^−06^	0.00077412
*CACNA1H*	−4.7419621	2.25 × 10^−29^	1.04 × 10^−17^	3.37 × 10^−6^
*CCDC109B*	2.08593613	4.67 × 10^−8^	2.73 × 10^−09^	0.00018476
*EFCAB4B*	2.03186213	4.66 × 10^−11^	1.96 × 10^−14^	4.98 × 10^−5^
*EFHA1*	0.74675824	0.00147429	0.00014479	0.00278932
*ITPR1*	0.83887283	3.72 × 10^−5^	0.00927132	0.00956689
*ITPR2*	1.76953569	4.12 × 10^−46^	5.79 × 10^−21^	3.01 × 10^−6^
*ITPR3*	0.52236077	3.25 × 10^−5^	0.0068986	0.01284295
*MCOLN1*	1.79737402	8.88 × 10^−82^	2.08 × 10^−17^	8.54 × 10^−7^
*MCU*	0.73588616	6.87 × 10^−8^	0.00151019	0.00278932
*MICU1*	0.50129943	0.00011815	0.02325149	0.01284295
*ORAI2*	2.15394804	4.74 × 10^−15^	3.48 × 10^−14^	0.00018476
*PKD2L1*	5.34354792	1.49 × 10^−8^	2.43 × 10^−10^	3.01 × 10^−6^
*RYR2*	12.5085599	1.17 × 10^−30^	1.39 × 10^−77^	2.95 × 10^−10^
*SLC8A2*	4.76101144	2.08 × 10^−15^	1.17 × 10^−14^	0.00010438
*STIM1*	1.92340546	5.41 × 10^−70^	1.20 × 10^−23^	1.80 × 10^−6^
*TRPA1*	−7.2445088	9.71 × 10^−8^	4.32 × 10^−10^	3.59 × 10^−6^
*TRPM5*	1.57085974	7.99 × 10^−6^	0.00121578	0.00097882
*TRPV6*	3.55456363	7.99 × 10^−6^	1.29 × 10^−6^	0.02627047
*VDAC1*	1.70533848	1.03 × 10^−75^	1.02 × 10^−39^	1.03 × 10^−8^
*VDAC2*	0.32791712	0.00017495	0.035753	0.00617185
*VDAC3*	1.50237461	6.80 × 10^−10^	1.07 × 10^−14^	5.86 × 10^−6^

## Supplementary Materials

Supplementary materials can be found at www.mdpi.com/1422-0067/18/5/922/s1.

## Abbreviations

CRC	Colorectal cancer
VOCC	Voltage-operated Ca^2+^ channels
SOCE	Store-operated Ca^2+^ entry
TRP	Transient receptor potential channels
IP_3_R	Inositol trisphosphate receptor
RyR	Ryanodine receptor
PMCA	Plasma membrane Ca^2+^/ATPase
SERCA	Sarcoplasmic and endoplasmic reticulum Ca^2+^/ATPase
SPCA	Secretory pathway Ca^2+^/ATPase
MCU	Mitochondrial calcium uniporter
